# Investigation of Effects of Ultrasound Therapy on Trapezius Muscle Stiffness and Choroidal Blood Flow Velocity

**DOI:** 10.3390/muscles5020028

**Published:** 2026-04-21

**Authors:** Takanori Taniguchi, Ryoutarou Mutou, Kokoro Oki, Miki Yoshimura, Yuko Kodama, Nao Nakamura, Yuki Hashimoto

**Affiliations:** 1 Department of Physical Therapy, Faculty of Medicine, Fukuoka International University of Health and Welfare, Momochihama 3-6-40, Sawara-ku, Fukuoka 814-0001, Japan; taniguchi.tg@ihwg.jp; 2 Department of Orthoptics, Faculty of Medicine, Fukuoka International University of Health and Welfare, Momochihama 3-6-40, Sawara-ku, Fukuoka 814-0001, Japan; 2313037@s.takagigakuen.ac.jp (R.M.); 2313008@s.takagigakuen.ac.jp (K.O.); 2213017@s.takagigakuen.ac.jp (Y.K.); 2213026@s.takagigakuen.ac.jp (N.N.); 3 Department of Orthoptics and Visual Sciences, Faculty of Health and Medical Sciences, Graduate School of Health and Welfare Sciences, International University of Health and Welfare, Akasaka 4-1-26, Minato-ku, Tokyo 107-8402, Japan

**Keywords:** autonomic regulation, choroidal blood flow, muscle stiffness, trapezius muscle, ultrasound therapy

## Abstract

This study evaluated changes in upper trapezius muscle stiffness and choroidal blood flow velocity before and after ultrasonic therapy of the trapezius muscle. Participants included 27 healthy young adults in their 20 s (median age [Q1–Q3]: 21.0 [19.3–21.0]) without subjective shoulder pain. All participants received a single-session ultrasound intervention, and no control group was included. Intraocular pressure (IOP), systolic blood pressure (BP), diastolic BP, mean BP, heart rate (HR), ocular perfusion pressure (OPP), and salivary α-amylase (sAA) activity, a marker of autonomic nerve function, were assessed at baseline and after therapy. Stiffness of the upper trapezius muscle was evaluated using shear wave elastography, and choroidal hemodynamics were assessed by measuring the mean blur ratio (MBR), a relative index of macular blood flow velocity, using laser speckle flowgraphy. IOP, systolic BP, diastolic BP, mean BP, HR, OPP, sAA activity, and MBR reduced significantly after therapy. The shear elastic modulus of the trapezius muscle also decreased significantly. However, no significant correlations were observed among the parameters. Among healthy adults in their 20 s without shoulder pain, trapezius muscle ultrasound therapy may enhance parasympathetic activity, contributing to decreases in systemic and choroidal circulatory parameters. These findings indicate that ultrasound therapy for shoulder stiffness may influence local musculoskeletal characteristics, systemic and ocular circulation, and autonomic pathways.

## 1. Introduction

In contemporary society, prolonged desk work and the widespread use of smartphones and computers have increased the number of individuals reporting neck and shoulder stiffness as well as muscle tension [1,2]. These lifestyle-related factors often involve sustained static postures and repetitive low-intensity muscle activity, which place continuous mechanical and metabolic demands on the cervical and shoulder musculature. In occupational settings, particularly among desk-based workers, such prolonged muscle loading has been recognized as a major contributor to musculoskeletal discomfort. Brandt et al. demonstrated a significant association between self-reported neck and shoulder pain and trapezius muscle tenderness, suggesting that sustained muscle activation combined with insufficient recovery plays a key role in the development and persistence of neck–shoulder pain in office workers [1]. Their findings highlight the importance of local muscle tenderness as an indicator of chronic muscle overload and impaired recovery processes. Similarly, Maayah et al. reported that prolonged smartphone use among university students was significantly associated with an increased prevalence and severity of neck pain, which was attributed to sustained neck flexion and altered head posture during device use [2]. These results suggest that both occupational and recreational screen-based activities contribute to excessive loading of the neck and shoulder muscles across different age groups. Importantly, excessive muscle tension in the neck–shoulder region cannot be explained solely by mechanical overload alone, as multiple physiological and behavioral appear to contribute. Psychological stress may sustain chronic muscle tension through prolonged low-level activation of postural muscles [3], while impaired endurance and activation of the deep cervical flexor muscles can further exacerbate neck pain [4]. At the tissue level, microtrauma to cervical structures may trigger protective muscle contraction (muscle guarding) via local inflammatory responses [5]. Behaviorally, asymmetric loading during dominant-side activities promotes sustained low-intensity muscle activity and local fatigue [6], and physical inactivity has similarly been associated with neck pain [7]. In addition, abnormal breathing patterns, particularly excessive use of accessory respiratory muscles, have been linked to increased activity of the neck–shoulder muscles [8]. Taken together, muscle stiffness in this region reflects a multifactorial phenomenon involving complex interactions among various factors. Shoulder stiffness is characterized by elevated muscle hardness resulting from reduced intramuscular blood flow and the accumulation of metabolic byproducts, such as lactate, which together contribute to pain, discomfort, and muscular fatigue that interfere with daily activities. Sustained static contraction of the neck and shoulder muscles has been shown to further impair local circulation and increase muscle activity, thereby exacerbating these symptoms. Such an ischemic environment has been shown to enhance anaerobic metabolism and promote the accumulation of metabolic by-products, thereby sensitizing nociceptors through alterations in the intramuscular milieu [9]. Moreover, the local accumulation of algogenic substances, including bradykinin, substance P, and prostaglandins, directly stimulates nociceptors within muscle tissue [10] and induces protective muscle contraction [11]. Central sensitization in chronic pain conditions is also characterized by increased excitability of dorsal horn neurons in the spinal cord [12], which presumably contributes to hyperalgesia and augmented muscle tone. Hallman et al. demonstrated that static trapezius muscle contraction significantly alters trapezius blood flow and is accompanied by changes in cardiovascular autonomic indices, indicating a close interaction between local muscular load and autonomic nervous system regulation in individuals with chronic neck–shoulder pain [13]. Moreover, excessive tension in the neck and shoulder musculature is closely linked to heightened sympathetic nervous system activity. Prolonged sympathetic activation not only affects cardiovascular regulation but may also have broader systemic consequences. Using 24 h ambulatory monitoring, it was reported that individuals with musculoskeletal pain exhibit altered autonomic regulation characterized by increased sympathetic dominance and reduced parasympathetic activity, which was further associated with perceived stress levels and physical activity patterns [14]. Such autonomic imbalances may influence both peripheral and central blood flow regulation, suggesting potential effects on systemic and ocular circulation through shared autonomic pathways.

Eye strain and visual discomfort are closely linked to shoulder stiffness and neck–shoulder discomfort, particularly during visually demanding near work [15]. Zetterberg et al. demonstrated that neck and shoulder discomfort induced by sustained near-vision tasks is influenced not only by task duration and previous neck pain, but also by visual factors such as astigmatism, accommodative demand, and internal eye discomfort [15]. These findings suggest that visual load and musculoskeletal strain are closely interconnected, potentially through shared neural and physiological mechanisms rather than acting as independent factors.

One possible mechanism linking shoulder stiffness and visual discomfort is autonomic regulation of ocular circulation, particularly choroidal hemodynamics. The choroid is a highly vascularized tissue that plays a crucial role in supplying oxygen and nutrients to the outer retina, including the photoreceptors. In contrast to the retinal circulation, which exhibits relatively strong autoregulatory capacity, choroidal blood flow is known to be more susceptible to systemic and neural influences [16].

Previous physiological studies have shown that choroidal blood flow is strongly regulated by the autonomic nervous system, with both sympathetic and parasympathetic innervation contributing to vascular tone modulation [17]. Sympathetic activation generally induces vasoconstriction, whereas parasympathetic input promotes vasodilation. Nickla and Wallman further emphasized the multifunctional nature of the choroid, highlighting its roles not only in metabolic support of the retina but also in thermoregulation, emmetropization, and accommodation-related responses [18]. Given this limited autoregulatory capacity and strong autonomic control, choroidal circulation may be particularly vulnerable to systemic autonomic imbalance.

Taken together, these findings suggest that excessive neck and shoulder muscle tension, through heightened sympathetic nervous system activity, may influence ocular blood flow regulation and contribute to visual discomfort and eye strain. This autonomic linkage provides a plausible physiological pathway connecting musculoskeletal load in the neck–shoulder region with alterations in choroidal hemodynamics, underscoring the importance of investigating ocular circulation in individuals with shoulder stiffness. Previous studies have reported that choroidal blood flow velocity is sensitive to changes in autonomic nervous system activity and increases under conditions of sympathetic dominance, such as acute stress or neck and shoulder muscle tension, as assessed by laser speckle flowgraphy (LSFG) [19,20]. Imabayashi et al. demonstrated that cold stimulation tests, which induce sympathetic activation via cold-induced stress and nociceptors, significantly alter choroidal hemodynamics in young healthy participants and increase choroidal blood flow velocity as measured by LSFG [19]. These findings provide direct evidence that choroidal circulation rapidly responds to systemic autonomic changes even in the absence of ocular pathology.

In addition to stress-induced autonomic activation, musculoskeletal factors have also been shown to influence choroidal circulation. Yoshimura et al. reported that self-stretching of the trapezius muscle led to a reduction in muscle stiffness accompanied by measurable changes in choroidal circulatory dynamics, as evaluated using ultrasound strain elastography and LSFG [20]. Their results suggest that alleviation of neck and shoulder muscle tension can modulate autonomic balance and, consequently, affect choroidal blood flow. This interaction highlights a physiological link between musculoskeletal condition and ocular circulation mediated by autonomic pathways.

Therapeutic ultrasound is widely used in clinical and rehabilitation settings to address muscle hypertonicity, reduced tissue extensibility, and circulatory insufficiency. It delivers high-frequency sound waves, typically in the range of 1–3 MHz, which penetrate deep tissues and induce both thermal and mechanical effects [21]. Morishita et al. demonstrated that therapeutic ultrasound significantly increases intramuscular blood circulation and improves oxygen dynamics within skeletal muscle, indicating its effectiveness in enhancing local perfusion and metabolic activity [21]. Such improvements in microcirculation are considered essential for alleviating ischemia-related muscle stiffness and promoting tissue recovery. The thermal effects of therapeutic ultrasound elevate tissue temperature, leading to vasodilation, increased local blood flow, enhanced enzymatic activity, and improved muscle viscoelastic properties. These changes facilitate muscle relaxation and reduce passive stiffness, thereby improving flexibility and decreasing discomfort. In addition to thermal effects, ultrasound produces mechanical microvibrations that exert non-thermal influences on biological tissues. Robertson and Baker reviewed evidence suggesting that these mechanical effects may alter cell membrane permeability, enhance ion exchange, and promote tissue healing, ultimately contributing to reductions in inflammation and pain [22]. Furthermore, psychological and physical stress are associated with a state of sympathetic nervous system predominance, which has been reported to be related to reduced muscle blood flow and increased muscle stiffness [3,13,14]. Thermal stimulation induced by therapeutic ultrasound may modulate autonomic nervous system activity, thereby attenuating sympathetic predominance and contributing to a reduction in stress-related muscle tension. From the perspective of muscle imbalance, decreased strength of the deep cervical flexor muscles is often accompanied by compensatory overactivity of superficial muscles, such as the sternocleidomastoid and upper trapezius, resulting in sustained muscle tension and local ischemia [4]. Therapeutic ultrasound may temporarily reduce muscle tension in these overactive muscles and alleviate pain or discomfort. In addition, following trauma or repetitive microtrauma, local inflammation and protective muscle contraction may occur, leading to chronic increases in muscle tension [5]. Therapeutic ultrasound may contribute to the attenuation of such protective muscle contractions by enhancing tissue permeability and modulating the tissue repair process. Through these combined mechanisms, therapeutic ultrasound has been shown to be effective in decreasing muscle hardness and alleviating pain associated with shoulder stiffness.

Recent research has increasingly employed muscle stiffness as a quantitative marker of muscle function, fatigue, and pathological muscle conditions. Changes in muscle stiffness have been associated with pain, functional impairment, and altered neuromuscular control, making it a clinically relevant parameter for assessing musculoskeletal health. Ultrasound elastography provides an objective and noninvasive method for assessing muscle stiffness by visualizing and quantifying tissue elasticity in real time [23,24]. Oglat and AbuKhalil reviewed the fundamental principles of ultrasound elastography and highlighted its utility in evaluating soft tissue mechanical properties across a wide range of clinical applications, including musculoskeletal assessment [23]. Similarly, Sigrist et al. described various elastography techniques and emphasized their advantages in providing quantitative, reproducible measures of tissue stiffness that complement conventional B-mode ultrasound imaging [24].

This study aimed to investigate the effects of therapeutic ultrasound applied to the trapezius muscle on muscle stiffness and choroidal hemodynamics using ultrasound elastography and LSFG. Specifically, this study evaluated changes in trapezius muscle stiffness and corresponding alterations in choroidal blood flow to determine whether ultrasound therapy for shoulder stiffness influences ocular circulation. Furthermore, this study aimed to clarify whether modulation of muscle stiffness through ultrasound therapy contributes to systemic and ocular circulatory regulation via autonomic nervous system mechanisms.

We hypothesized that therapeutic ultrasound would reduce trapezius muscle stiffness, improve regional blood flow, and attenuate sympathetic nervous system activity, resulting in measurable changes in choroidal hemodynamics. We further hypothesized that the degree of reduction in muscle stiffness would be significantly associated with changes in choroidal blood flow parameters, supporting a mechanistic link between musculoskeletal relaxation and ocular circulatory regulation.

## 2. Results

**Figure 1:**
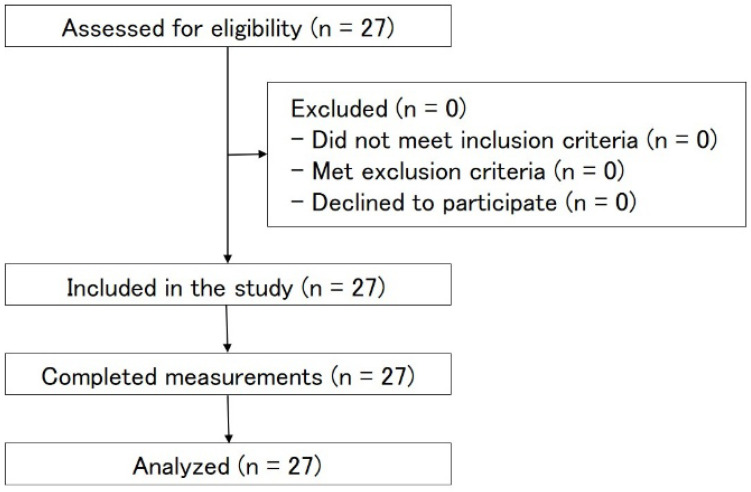
Flow diagram of participant selection. All individuals assessed for eligibility met the inclusion criteria. No participant was excluded or declined to participate.

Figure 1 shows the participant selection process. No participant was excluded or declined to participate. The median spherical equivalent refractive error was −2.13 [−3.50–0.00] diopters (D), with a range from +2.50 to −9.62 D, reflecting a broad distribution of refractive status among participants.

### 2.1. Intraocular Pressure, Blood Pressure, Salivary α-Amylase, and Visual Analog Scale

**Figure 2:**
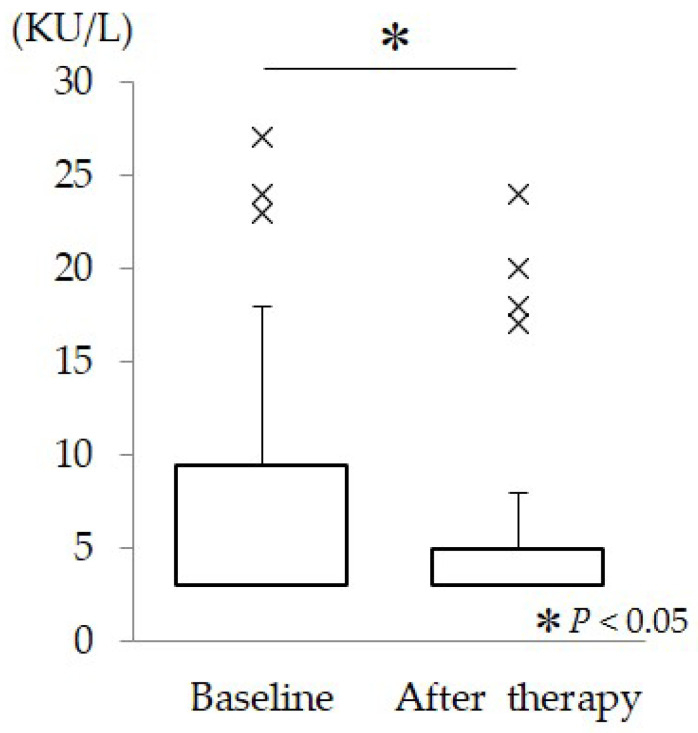
Boxplots showing salivary α-amylase (sAA) levels before and after ultrasound therapy. sAA levels were significantly lower after ultrasound therapy compared with baseline. The crosses (×) represent outliers, which are individual data points that fall outside the whiskers of the box plot.

Changes in intraocular pressure (IOP), systolic blood pressure (SBP), diastolic blood pressure (DBP), mean blood pressure (MBP), heart rate (HR), ocular perfusion pressure (OPP), and salivary alpha-amylase (sAA) activity are summarized in Table 1. Compared with baseline measurements, IOP decreased significantly after therapy (*p* = 0.004). In addition, SBP (*p* < 0.001), DBP (*p* = 0.025), and MBP (*p* < 0.001) all showed significant reductions, indicating a systemic decrease in blood pressure following the intervention. HR was also significantly reduced (*p* = 0.029), suggesting attenuation of cardiovascular sympathetic activity. Correspondingly, OPP decreased significantly after therapy (*p* = 0.005). Furthermore, sAA activity, a biomarker of sympathetic nervous system activation, showed a significant decrease compared with baseline values (*p* = 0.023) (Figure 2). This reduction in sAA activity suggests that the intervention induced a shift toward reduced sympathetic dominance. Baseline visual analog scale (VAS) scores for subjective symptoms were low but detectable, with shoulder pain, eye strain, and irritability scores of 0.8 ± 0.1 cm, 2.7 ± 0.4 cm, and 0.7 ± 0.3 cm, respectively. After therapy, VAS scores decreased significantly to 0.4 ± 0.1 cm for shoulder pain, 1.5 ± 0.3 cm for eye strain, and 0.4 ± 0.1 cm for irritability (*p* < 0.001, *p* = 0.001, and *p* < 0.001, respectively), indicating a consistent reduction in subjective discomfort and stress-related symptoms.

### 2.2. Mean Blur Rate

**Figure 3:**
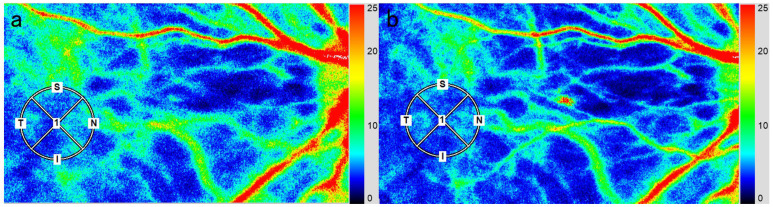
Laser speckle flowgraphy images of a healthy adult participant at baseline (**a**) and after upper trapezius ultrasound therapy (**b**) in case 3. The circle used to acquire the mean blur rate (MBR) was centered on the macula. The MBR decreased by 10.7% after ultrasound therapy (**b**) compared with baseline values (**a**). Blue indicates low MBR, and red indicates high MBR.

Mean blur rate (MBR) measurements obtained by LSFG are presented in Table 1 and Figure 3. The median baseline MBR value was 13.0 [9.6–17.0] (Figure 3a), whereas the post-therapy value decreased to 11.6 [9.5–15.7] (Figure 3b). This corresponded to a significant relative decrease of −9.7 [−15.5–−3.8]% compared with baseline (*p* < 0.001). These findings demonstrate that therapeutic intervention was associated with a significant reduction in choroidal blood flow velocity, reflecting altered ocular hemodynamics following muscle relaxation and autonomic modulation. Intraclass correlation coefficients (ICCs) demonstrated excellent reproducibility, with an ICC (1,1) of 0.95 (95% confidence interval [CI], 0.87–0.98) and an ICC (1,3) of 0.99 (95% CI, 0.95–0.99). These results confirm the robustness and reliability of LSFG for quantifying changes in MBR in this study.

### 2.3. Stiffness of the Trapezius Muscle

**Figure 4:**
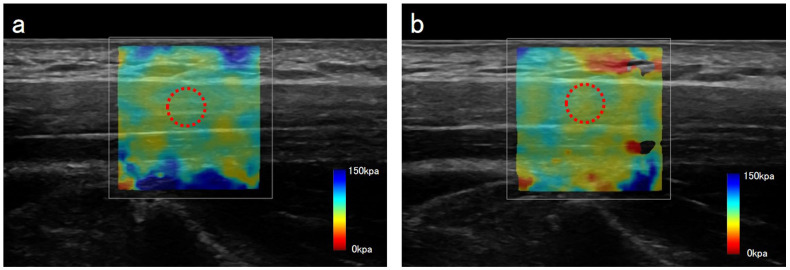
Shear wave elastography images of a healthy adult participant at baseline (**a**) and after upper trapezius ultrasound therapy (**b**). The color map is set such that blue indicates high trapezius stiffness and red indicates low trapezius stiffness. The region of interest was adjusted to muscle thickness, and a central circular Q-box was placed.

Changes in trapezius muscle stiffness assessed by shear wave elastography are shown in Table 1 and Figure 4. The baseline shear elastic modulus of the trapezius muscle was 77.5 [46.9–93.5] kPa, which significantly decreased to 46.2 [38.5–71.6] kPa after therapy (*p* = 0.001). This marked reduction indicated a substantial decrease in muscle stiffness following the intervention. The reliability of trapezius muscle stiffness measurements was high. ICCs demonstrated excellent reproducibility, with an ICC (1,1) of 0.95 (95% CI, 0.87–0.98) and an ICC (1,3) of 0.99 (95% CI, 0.95–0.99). These results confirm the robustness and reliability of ultrasound elastography for quantifying changes in muscle stiffness in this study.

### 2.4. Correlation Between MBR and Stiffness of the Trapezius Muscle or sAA

The change rate in MBR after ultrasound therapy showed no significant correlation with changes in trapezius muscle stiffness or sAA (*R* = −0.098, *p* = 0.623 and *R* = −0.062, *p* = 0.757, respectively).

## 3. Discussion

This study demonstrated that therapeutic ultrasound applied to the trapezius muscle significantly reduced muscle stiffness, subjective symptoms, systemic hemodynamic parameters, sAA activity, and choroidal blood flow velocity. These findings indicate that ultrasound therapy, although traditionally regarded as a localized intervention for relieving muscle stiffness, may exert broad physiological effects encompassing not only the autonomic nervous system but also the systemic and ocular region. The observed simultaneous changes in musculoskeletal, cardiovascular, autonomic, and ocular parameters suggest an integrated response extending beyond the treated muscle tissue.

Although few studies have reported the effects of ultrasound therapy on trapezius muscle stiffness, the results of the present study are consistent with those reported by Yildirim et al. [25]. The marked reduction in trapezius muscle stiffness following ultrasound therapy reflects the combined thermal and mechanical effects of high-frequency ultrasound energy. Thermal effects induce deep tissue heating, which enhances local blood flow, increases tissue extensibility, and reduces muscle viscosity, thereby facilitating relaxation of hypertonic muscle fibers [26,27]. These effects are consistent with established physiological mechanisms whereby elevated intramuscular temperature improves the viscoelastic properties of collagen-rich tissues and decreases passive resistance. In addition, mechanical microvibrations generated by ultrasound may stimulate mechanotransduction pathways and alter cell membrane permeability, potentially reducing intramuscular pressure and nociceptive signaling. These mechanisms likely contribute to reducing tonic muscle activity and improving muscle compliance [28].

In addition, impaired muscle blood flow caused by increased muscle stiffness is thought to partially reflect excessive activation of the sympathetic nervous system [29]. Morikawa et al. reported that changes in trapezius muscle stiffness also influence autonomic nervous system activity [30]. This study found that ultrasound therapy reduced trapezius muscle stiffness and decreased sympathetic nerve activity, but did not demonstrate a significant correlation. Thus, whether ultrasound therapy regulates autonomic nervous system balance beyond its localized mechanical and thermal effects on muscle tissue remains unclear. Furthermore, in the present study, reductions in salivary α-amylase (sAA) activity, blood pressure, heart rate, and MBR after ultrasound therapy were observed. Salivary α-amylase is widely recognized as a sensitive and noninvasive biomarker of sympathetic nervous system activity and has been shown to correlate with plasma norepinephrine responses during acute stress [31]. Therefore, the significant decrease in sAA activity suggests attenuation of sympathetic arousal. Previous studies have similarly demonstrated that interventions promoting parasympathetic predominance, such as periocular skin warming, result in systemic autonomic changes accompanied by alterations in choroidal circulation [32]. Moreover, Kuwahara et al. reported that parasympathetic dominance is associated with a reduction in choroidal blood flow velocity as measured by laser speckle flowgraphy [33]. However, sAA activity is influenced not only by sympathetic nerve stimulation but also by circadian rhythms, food intake, physical exercise, emotional arousal, parasympathetic innervation of the salivary glands, and salivary flow rate [34]. Previous methodological critiques have also noted that although the correlation between sAA and plasma norepinephrine is statistically significant at the population level, it is weak at the individual level [35]. Moreover, the response of sAA to sympathetic stimulation is highly context-dependent [35]. In this study, measurement conditions were standardized by considering circadian variation, with measurements performed between 15:00 and 21:00, and participants were instructed to refrain from smoking and exercise for at least 2 h prior to testing and rest for 10 min in a quiet room. However, the interpretation of sAA results in this study has limitations and should be approached with caution.

A particularly notable finding of this study was the significant reduction in MBR, a relative value of choroidal blood flow velocity measured by LSFG. The choroid possesses limited autoregulatory capacity compared with the retinal circulation and is therefore highly sensitive to autonomic modulation. While retinal blood flow remains relatively stable across systemic fluctuations, choroidal circulation responds rapidly to changes in sympathetic and parasympathetic activity. Previous studies have demonstrated that sympathetic activation increases choroidal blood flow velocity, whereas reduced sympathetic output or enhanced parasympathetic activity leads to decreased choroidal perfusion [19,22,23]. In addition, a prior report indicates that musculoskeletal interventions, such as trapezius muscle stretching, can alter autonomic balance and influence choroidal hemodynamics in healthy individuals [20]. Taken together, the results of this study highlight the need to further investigate the effects and relevance of ultrasound therapy applied to the trapezius muscle in relation to autonomic nervous system activity and systemic or ocular hemodynamics.

Despite its strengths, this study has some limitations that should be acknowledged. First, the study population consisted exclusively of young, healthy adults without clinically significant shoulder pain or diagnosed musculoskeletal disorders. Although this homogeneous cohort was advantageous for minimizing confounding factors, it may limit the generalizability of the findings to older individuals or patients with chronic shoulder stiffness, myofascial pain, or other musculoskeletal conditions. In such clinical populations, baseline muscle stiffness, pain perception, and autonomic regulation may differ substantially, potentially leading to different therapeutic responses. Second, the duration and temporal profile of the observed effects were not evaluated in this study. Measurements were obtained immediately after ultrasound therapy, and therefore, it remains unclear how long the reductions in muscle stiffness, autonomic activity, and choroidal hemodynamics persist. The lack of follow-up assessments precludes conclusions regarding the sustainability of these effects or their clinical relevance over time. Longitudinal studies incorporating repeated measurements at multiple time points in accordance with therapeutic ultrasound physiology studies would be necessary to determine whether the observed changes are transient or represent longer-lasting physiological adaptations. Third, choroidal evaluation was restricted to LSFG. Multimodal evaluation of choroidal morphology and circulation using enhanced depth imaging optical coherence tomography (OCT) or swept source-OCT (OCT) and OCT angiography, in addition to LSFG, would provide a more comprehensive understanding. In addition, only the right eye was analyzed. Although this approach was adopted to maintain statistical independence and avoid inter-eye correlation bias, bilateral measurements would provide additional confirmation of symmetric effects. Fourth, the baseline shear wave elastography (SWE) values of the upper trapezius reported in this study were higher than the normal range previously reported for asymptomatic adults (13.3–41.7 kPa). In the present study, participants were defined as “asymptomatic” based on the absence of shoulder pain requiring medical consultation and a VAS score of less than 3 cm. However, the median baseline VAS score was 0.8 cm, indicating that although no overt pain was reported, the presence of mild latent muscle tension or fatigue cannot be excluded. Taken together, these findings suggest that the study population may not represent a completely pain-free healthy cohort and should therefore be interpreted as limited to young adults without clinically significant shoulder pain. Fifth, the effects of time, repeated measurements, and other potential confounding factors could not be adequately excluded due to the lack of a control group. Future studies should include a control group to evaluate the effects of ultrasound therapy more rigorously. Sixth, in addition to the sympathetic nervous system, multiple mechanisms may contribute to trapezius muscle stiffness, including local hypoxia, central sensitization, and the accumulation of substances that stimulate nociceptors, such as bradykinin, substance P, and prostaglandins. However, in the present study, these factors were not directly assessed or measured, and their individual contributions could not be evaluated separately. Finally, although multiple indirect indicators of autonomic nervous system activity, including heart rate, blood pressure, and salivary α-amylase activity, were assessed, direct measurements of autonomic nerve activity were not obtained. While these surrogate markers are widely used and provide valuable insight into autonomic balance, more direct approaches, such as heart rate variability spectral analysis or microneurography, could offer a more comprehensive understanding of sympathetic and parasympathetic modulation. Incorporating such measures in future studies may help clarify the precise autonomic mechanisms underlying the observed musculoskeletal and ocular circulatory changes.

## 4. Materials and Methods

### 4.1. Participants

This study was approved by the Ethics Committee of Fukuoka International University of Health and Welfare (approval ID: 25-TG-017) and was conducted in strict accordance with the principles of the Declaration of Helsinki and its subsequent amendments. Before participation, all individuals received a detailed explanation of the study objectives, procedures, potential risks, and benefits, and written informed consent was obtained from each participant. This prospective pre–post intervention study included the right eyes of 27 university students (14 females and 13 males). The median age of the participants was 21.0 [19.3–21.0] years (range, 18–23 years). None reported subjective shoulder pain or clinically significant musculoskeletal symptoms at baseline, indicating a relatively homogeneous young adult cohort. We only included healthy adults without clinical neck or shoulder symptoms to clarify the isolated physiological effects of ultrasound therapy on muscle and ocular function. Muscle activity patterns in individuals experiencing pain or discomfort may be influenced by pain-related factors such as pain-avoidant behavior, alterations in motor control, and central sensitization, which may confound result interpretation and hinder accurate evaluation of the specific effects of ultrasound therapy. To maintain statistical independence and avoid inter-eye correlation, only the right eye of each participant was analyzed. Participants were recruited using the voluntary response type of non-probability sampling. Participants were excluded if they had a history of systemic, ophthalmic, orthopedic, or neurological diseases that could influence ocular circulation, autonomic nervous system function, or musculoskeletal properties. Additional exclusion criteria included the inability to undergo any of the required examinations, poor fixation or cooperation during ocular measurements, and the inability to provide informed consent. Individuals taking medications known to affect autonomic function, blood pressure, or ocular blood flow were also excluded to reduce potential confounding effects.

Participants were recruited through voluntary response following an explanation of the study purpose and procedures. No financial incentives were provided, and participation was entirely optional. All measurements were conducted in a controlled laboratory environment under standardized conditions to minimize external influences. Specifically, they were performed in a quiet examination room at room temperature (24 ± 1 °C), with humidity maintained at 47 ± 3%. In addition, all measurements were performed between 13:00 and 21:00 to minimize the potential influence of diurnal variation. Furthermore, participants were asked to refrain from smoking and exercise for at least 2 h prior to testing and were permitted a 10 min rest period in a quiet room. Each participant underwent a comprehensive ophthalmic and physiological assessment protocol. This included objective refraction testing and measurement of best-corrected visual acuity to ensure accurate evaluation of visual function. IOP, BP, and HR were measured to assess ocular and cardiovascular status. Autonomic nervous system activity was evaluated using sAA in addition to BP and HR. Subjective symptoms of shoulder stiffness, eye strain, and related discomfort were assessed using a standardized VAS. In addition, fundus photography and LSFG were performed to evaluate ocular structure and choroidal hemodynamics, respectively. Finally, shear wave elastography was used to quantitatively assess trapezius muscle stiffness before and after the intervention.

Baseline measurements were performed in the following order: VAS, LSFG, BP and HR, IOP, sAA, and SWE. Moreover, immediately after ultrasound therapy, examinations were conducted in the same order as baseline. Standardizing conditions for all participants minimized the influence of examination order and examination-related effects on results. All measurements were taken in a seated position. Each experimental session (VAS: 60 s, LSFG: 60 s, BP and HR: 90 s, IOP: 30 s, sAA: 30 s, SWE: 120 s) was completed within 7 min.

### 4.2. IOP and Systemic Hemodynamics

IOP, SBP, DBP, and HR were assessed before and after trapezius ultrasound therapy. IOP was measured with a non-contact tonometer (NT-530, NIDEK Co., Ltd., Aichi, Japan). BP was measured using an automatic BP monitor (Omron, OMRON DALIAN Co., Ltd., Kyoto, Japan). MBP was calculated from SBP and DBP (1), and OPP was calculated from MBP and IOP (2) [36,37].

(1) MBP=DBP+1/3(SBP-DBP)

(2) OPP=2/3(MBP-IOP)

### 4.3. Autonomic Nervous System Assessment

sAA activity was measured using a salivary amylase monitor (Nipro Co., Ltd., Osaka, Japan). This noninvasive index reflects plasma norepinephrine concentration and sympathetic nervous system reactivity. The saliva collection paper was placed under the tongue for 30 s, and sAA activity was recorded.

### 4.4. Subjective Symptoms of Shoulder Stiffness, Eye Strain, and Irritability

Subjective symptoms were assessed using a VAS developed for this study. Participants recorded scores on a 10 cm line, with 0 indicating no symptoms and 10 indicating the most severe symptoms. Scores were measured with a 10 cm ruler. Cases in which VAS-measured shoulder pain was less than 3 cm were classified as having no shoulder pain.

### 4.5. LSFG

LSFG-NAVI (Softcare Ltd., Fukuoka, Japan) was used to assess posterior fundus hemodynamics. This system employs an 830 nm diode laser to illuminate the fundus and detect red blood cell movement in deep choroidal vessels [38,39]. The origin of the MBR is considered to be 92% choroidal and 8% retinal [28]. Furthermore, as the 400 µm diameter fovea is an avascular area, no retinal vessels are present. Therefore, LSFG could be a suitable method for monitoring changes in the choroidal circulation. The laser speckle technique provides a quantitative and reproducible assessment of blood flow velocity [40,41], with each acquisition completed in approximately 4 s. Measurements were obtained three times at baseline and after ultrasound therapy. Large retinal vessels in the macular region were excluded. For each participant, the same region of interest was automatically selected by the LSFG Analyzer software (version 3.0.47; Softcare Ltd., Fukuoka, Japan), ensuring consistency with the baseline site (Figure 3). Changes in average MBR, a relative index of blood flow velocity, were evaluated as the percentage relative to baseline (set as 100%).

### 4.6. Shear Wave Elastography

Trapezius muscle stiffness was assessed using the shear wave elastography function of an ultrasonic diagnostic system (LOGIQ P9; GE Healthcare, Tokyo, Japan). Each participant sat upright with the forearm relaxed and the palm facing upward on the ipsilateral thigh to maximize trapezius relaxation. Based on previous reports, the measurement site was the midpoint between the 7th cervical spinous process and the posterior acromial corner [42]. A linear probe was aligned with the muscle fibers. The trapezius muscle was first visualized in B-mode, after which shear wave elastography mode was activated to define the elasticity region. Probe pressure was minimized, and data were collected when the shear wave elastography display stabilized. The region of interest was adjusted to muscle thickness, and a central circular Q-box was placed. The shear elastic modulus within the Q-box was measured three times, and the mean value was used for analysis [43]. All assessments were performed by the same examiner. Ultrasound elastography images from 10 randomly selected participants were reanalyzed in random order, and intraobserver reproducibility was evaluated using the ICC.

### 4.7. Ultrasound Therapy

Ultrasound therapy was performed using a therapeutic ultrasound device (PHYSIO SONO; SAKAI med, Tokyo, Japan). Previous research indicates that continuous 3 MHz ultrasound at 2.0 W/cm^2^ rapidly elevates muscle temperature [44], and the subsequent decline in temperature is gradual, providing sustained thermal stimulation [45]. Therefore, ultrasound settings were 3 MHz frequency, 2.0 W/cm^2^ intensity, 100% duty cycle (continuous mode), a transducer head movement speed of 1 cm/s, and 10 min of irradiation using the stroke method. The treatment area was approximately twice the effective radiating area and centered on the site where trapezius elasticity had been measured. Ultrasound therapy was a single session.

### 4.8. Statistical Analyses

The primary endpoints were predefined as trapezius muscle stiffness and MBR, with approval obtained from the institutional ethics committee prior to study initiation. Secondary outcomes included IOP, SBP, DBP, MBP, HR, OPP, sAA, and VAS scores. The Wilcoxon signed-rank test was used to evaluate pre–post changes in IOP, SBP, DBP, HR, MBP, OPP, sAA activity, VAS scores for subjective symptoms, trapezius muscle stiffness, and MBR before and after trapezius ultrasound therapy. This nonparametric test was selected because several outcome variables did not satisfy the assumptions of normality, and the study employed a within-subject repeated-measures design. Spearman’s rank correlation coefficient was used to examine the correlations of the MBR change rate with changes in trapezius muscle stiffness and sAA. In addition, because three highly myopic eyes (≤−6.00 D) were included in this study, Spearman’s correlation analysis was also performed to examine the relationship between spherical equivalent refractive error and MBR values to evaluate the influence of refractive error on MBR. All statistical tests were two-tailed, and a *p*-value of less than 0.05 was considered to indicate statistical significance. Statistical analyses were performed using BellCurve for Excel (version 4.08; Social Survey Research Information Co., Ltd., Tokyo, Japan), a validated statistical software package commonly used in biomedical research. Descriptive statistics are presented as the median [Q1–Q3] to appropriately reflect the distribution of the data and to provide robust estimates of central tendency and variability.

## 5. Conclusions

In conclusion, in this single-arm pre–post study of healthy young adults without pain complaints, significant reductions in trapezius muscle stiffness, subjective discomfort, systemic hemodynamic parameters, sAA, and choroidal blood flow velocity were observed immediately after a single therapeutic ultrasound session. These concurrent changes indicate that multiple physiological parameters changed following the intervention; however, because the study lacked a control group and assessed only short-term outcomes, no causal inferences can be drawn. Thus, these findings should be considered preliminary and are limited to young adults without clinically significant shoulder disorders. Furthermore, the observed changes may reflect nonspecific physiological responses involving central nervous system regulation and circulating chemical mediators, such as vasoactive or stress-related factors, in addition to any effects specific to ultrasound therapy. Future studies employing research designs with appropriate control conditions, more diverse participant populations, and longitudinal assessments are needed to elucidate the mechanisms underlying these associations and to evaluate their clinical relevance in symptomatic populations.

## Figures and Tables

**Table 1:** Changes in biometric parameters and general eye factors before and after ultrasound therapy of the upper trapezius muscle in the participants.

	Baseline (Median [Q1–Q3])	After Ultrasound Therapy (Median [Q1–Q3])	*p* Value (Wilcoxon Signed-Rank Test)
IOP (mmHg)	14.3 [10.0–15.5]	13.3 [11.9–15.0]	0.004 **
SBP (mmHg)	116.0 [111.3–121.8]	109.0 [104.5–114.8]	<0.001 ***
DBP (mmHg)	73.5 [67.3–77.3]	70.5 [65.5–75.3]	0.025 *
MBP (mmHg)	87.2 [82.8–92.8]	82.2 [79.9–86.9]	<0.001 ***
OPP (mmHg)	45.1 [41.7–47.0]	43.1 [39.2–44.7]	0.005 **
HR (bpm)	74.0 [70.0–81.8]	70.0 [63.8–77.0]	0.029 *
sAA (KU/L)	3.0 [3.0–9.5]	3.0 [3.0–5.0]	0.023 *
VAS (cm) stiff shoulder	0.8 [0.4–1.5]	0.4 [0.1–1.1]	<0.001 ***
VAS (cm) eye strain	2.7 [1.2–3.7]	1.5 [0.3–2.6]	0.001 **
VAS (cm) irritability	0.7 [0.4–1.8]	0.4 [0.1–0.7]	<0.001 ***
MBR	13.0 [9.6–17.0]	11.6 [9.5–15.7]	<0.001 ***
MBR (%)	100 [100]	91.3 [84.7–96.0]	<0.001 ***
Shear elastic modulus (kPa)	77.5 [46.9–93.5]	46.2 [38.5–71.6]	0.001 **

IOP, intraocular pressure; SBP, systolic blood pressure; DBP, diastolic blood pressure; MBP, mean blood pressure; HR, heart rate; OPP, ocular perfusion pressure; sAA, salivary α-amylase activity; MBR, mean blur rate; kPa, kilopascal. Wilcoxon signed-rank test. Median [Q1–Q3]. * *p* < 0.05, ** *p* < 0.01, *** *p* < 0.001.

## Data Availability

The original findings presented in this study are included in this paper. Detailed inquiries should be directed to the corresponding author.

## Abbreviations

The following abbreviations are used in this manuscript:

DBP	diastolic blood pressure
HR	heart rate
ICC	intraclass correlation coefficient
IOP	intraocular pressure
LSFG	laser speckle flowgraphy
MBP	mean blood pressure
MBR	mean blur rate
OPP	ocular perfusion pressure
sAA	salivary α-amylase
SBP	systolic blood pressure
VAS	visual analog scale
